# Nanocomposite Materials with Poly(l-lactic Acid) and Transition-Metal Dichalcogenide Nanosheets 2D-TMDCs WS_2_

**DOI:** 10.3390/polym12112699

**Published:** 2020-11-16

**Authors:** Mohammed Naffakh, Miriam Fernández, Peter S. Shuttleworth, Ana M. García, Diego A. Moreno

**Affiliations:** 1 Escuela Técnica Superior de Ingenieros Industriales, Universidad Politécnica de Madrid (ETSII-UPM), José Gutiérrez Abascal 2, 28006 Madrid, Spain; miriam.fernandezgall@alumnos.upm.es (M.F.); ana.garcia.ruiz@upm.es (A.M.G.); diego.moreno@upm.es (D.A.M.); 2 Instituto de Ciencia y Tecnología de Polímeros (ICTP-CSIC), Juan de la Cierva 3, 28006 Madrid, Spain; peter@ictp.csic.es; 3 Facultad de Farmacia, Universidad de Castilla-La Mancha (FF-UCLM), Avda. Dr. José María Sánchez Ibañez s/n, E-02071 Albacete, Spain

**Keywords:** 2D-WS_2_, PLLA, nanomaterials, morphology, crystallization kinetics, biodegradation

## Abstract

Layered transition-metal dichalcogenides (TMDCs) based on tungsten disulfide nanosheets (2D-WS_2_) were introduced via melt processing into poly(l-lactic acid) (PLLA) to generate PLLA/2D-WS_2_ nanocomposite materials. The effects of the 2D-WS_2_ on the morphology, crystallization, and biodegradation behavior of PLLA were investigated. In particular, the non-isothermal melt-crystallization of neat PLLA and PLLA/2D-WS_2_ nanocomposites were analyzed in detail by varying both the cooling rate and 2D-WS_2_ loading. The kinetic parameters of PLLA chain crystallization are successfully described using the Liu model. It was found that the PLLA crystallization rate was reduced with 2D-WS_2_ incorporation, while the crystallization mechanism and crystal structure of PLLA remained unchanged in spite of nanoparticle loading. This was due to the PLLA chains not being able to easily adsorb on the WS_2_ nanosheets, hindering crystal growth. In addition, from surface morphology analysis, it was observed that the addition of 2D-WS_2_ facilitated the enzymatic degradation of poorly biodegradable PLLA using a promising strain of actinobacteria, *Lentzea waywayandensis*. The identification of more suitable enzymes to break down PLLA nanocomposites will open up new avenues of investigation and development, and it will also lead to more environmentally friendly, safer, and economic routes for bioplastic waste management.

## 1. Introduction

Poly(l-lactic acid) (PLLA) is a highly versatile, biodegradable, aliphatic polyester derived from 100% renewable resources, such as corn and sugar beets. This bioplastic offers great promise in a wide range of environmental and biomedical applications due to its favorable biodegradability, renewability, reasonably good mechanical properties, and versatile fabrication methods [1,2,3]. PLLA and its degradation products, namely H_2_O and CO_2_, are neither toxic nor carcinogenic to the human body, making it an excellent material for biomedical applications including sutures, clips, and drug delivery systems (DDS). Furthermore, PLLA can be processed by film casting, extrusion, blow molding, and fiber spinning due to its better thermal processability in comparison to other biomaterials such as poly(ethylene glycol) (PEG), poly(hydroxyalkanoates) (PHAs), and poly(ε-caprolactone) (PCL) [4]. In spite of its excellent balance of properties, its commercial viability has historically been limited by high production costs and poor crystallizability.

Recently, several PLLA-based nanotechnologies have emerged with an emphasis on achieving chemical, thermal, mechanical, and biological properties superior to conventional biopolymers, opening new possibilities for the plastic industry. However, since polylactic acid (PLA) is classified as a hard bio-polyester that is prone to hydrolysis, understanding and engineering of its thermo-mechanical properties and its nanocomposites are crucial for use in cutting-edge applications [5,6]. Along with many other interesting nanofillers, the use of layered transition-metal dichalcogenide nanostructures (TMDCs), such as molybdenum disulfide (MoS_2_) and tungsten disulfide (WS_2_), which are high-band gap semiconductors with 0D, 1D, and 2D structural anisotropy are particular interesting. As an emerging 2D layered nanomaterial, it has been recently reported that monolayer MoS_2_ with high surface area, superb thermal stability, and excellent mechanical properties [7] exhibits great potential as a reinforcement agent for polymers [8,9]. In addition, it has been shown that the 2D-TMDCs can potentially improve the polymeric materials mechanical and barrier properties, whilst not effecting their electrical insulation properties (e.g., polyurethane (PU) [8], polypropylene (PP) [10], poly(vinyl alcohol) (PVA) [11]. In particular, the use of environmentally friendly and biocompatible inorganic TMDCs have been shown to offer design, processing, performance, and cost advantages compared to carbon nanotubes, nanoclays, or other inorganic nanoparticles [12,13] when manufacturing advanced biopolymer nanocomposites (Bio-PNCs 1D-WS_2_) (poly(propylene fumarate) (PPF), poly(3-hydroxybutyrate) (PHB), poly(ether ether ketone) (PEEK), PLLA, etc.) [14,15,16,17]. More specifically, the thermo-mechanical properties of PLLA biopolymers are directly related to their biomedical performance when interfaced with biological systems, since these properties can be used to optimize important design criteria (e.g., modulus, strength, morphology, crystallinity, biocompatibility, etc.) and, in turn, these properties can affect cell response, tissue regeneration, and in vivo degradation. In addition, research shows that different thermal treatments affect not only the crystallinity of PLLA polymers but also of other bio-polyesters, which is a critical parameter for cell biocompatibility and drug release dynamics [18].

It is well known that the crystallization of polymers is complex and is affected by a variety of factors that include temperature, cooling rate, and flow-induced deformation as well as the size, shape, and volume fraction of additive nanoparticles. In particular, controlling the crystallinity of hybrid polymeric systems has an important impact on their properties and is essential for developing novel functional materials. Despite many experiments to understand the effect of nano-additives on crystallization, the results have often been contradictory. Therefore, the control of crystallinity in hybrid molecular systems remains empirical at best. Jabbarzadeh has recently investigated the origins of enhanced and retarded crystallization in nanocomposite polymers [19]. The results of large-scale molecular dynamics simulations revealed that while crystallinity was affected by the nanoparticle size and its volume fraction, their combined effects can only be measured by interparticle free space and the characteristic size of the crystals. Understanding the dynamics of these systems, including the mobilities of the different constituents, also remains an extremely difficult task, despite the wide-ranging research interest in them [20,21].

On the other hand, the study of biodegradability of biopolymer nanocomposite materials using laboratory-scale testing is extremely important from both an industrial and scientific perspective. Biodegradation can be influenced by many different factors, including biopolymer characteristics, the type of microoganism, and pre-treatment. Polymer characteristics, such as mobility, tacticity, crystallinity, molecular weight, chemical functionality, and substituents present in its structure, and plasticizers or nanoparticle additives added to the polymer all play an important role in its degradation. In addition, it has been reported [22,23] that adding hydrophilic nanoparticles can accelerate PLA biodegradation as water molecules can more easily penetrate into the polymeric matrix. However, other studies [24,25] have reported that biodegradation was retarded due to an enhancement in the nanocomposites barrier properties.

The aim of the current study is to demonstrate the advantages of using 2D-WS_2_ as a suitable nano-reinforcement to enhance PLLA performance. The nanocomposites were prepared via a versatile, economic, and scalable melt-processing route. In particular, the influence of the 2D-WS_2_ on the processability, morphology, biodegradation, and crystallization behavior of the resulting PLLA/2D-WS_2_ nanocomposites are analyzed.

## 2. Experimental Section

### 2.1. Materials and Processing

PLLA in granule form (density = 1.25 g/cm^3^, *M*_w_ ≈ 1.5 × 105 g/mol) was supplied by Goodfellow Ltd. (Huntingdon, UK) and used as received. The 2D-WS_2_ nanosheets (density ≈ 7.5 g/cm^3^, width/length ≈ 20–500 nm, and thickness ≈ 1 nm) were obtained from ACS Material LLC (Medford, MA, US) and used without chemical modification. To prepare the PLLA/2D-WS_2_ nanocomposites, PLLA and 2D-WS_2_ (0.1, 0.5 and 1.0 wt %) were dispersed together in a small volume of ethanol (HPLC grade, Sigma-Aldrich Química SL, Madrid, Spain) and homogenized by mechanical stirring and bath ultrasonication for approximately 10 min. Subsequently, the ethanol was evaporated off, and the PLLA/2D-WS_2_ dispersion was dried under vacuum at 60 °C, 70 mbar for 24 h. Melt-mixing of the resulting dispersions was performed using a micro-extruder (Thermo-Haake Minilab system) operated at 190 °C and a rotor speed of 100 rpm for 10 min [17]. Then, the samples were pressed into film thicknesses of 0.3–0.5 mm in a hot press system using two heating/cooling plates (Collin P-200, Collin Lab & Pilot Solutions GmbH, Maitenbeth, Germany).

### 2.2. Characterization Studies 

#### 2.2.1. Scanning Electron Microscopy (SEM)

The morphology of degradable and non-degradable samples was characterized using ultra-high field-emission scanning microscopes (FESEM), JEOL-JSM7600F, and SU8000-Hitachi Co., Ltd. (Tokyo, Japan), respectively. All specimens were sputter coated with gold or/and Au/Pd prior to analysis.

#### 2.2.2. Wide-Angle X-ray Diffraction (WAXS)

WAXS diffractograms were obtained using a Bruker D8 Advance diffractometer (Bruker AXS GmbH, Karlsruhe, Germany) employing Ni-filtered CuKα radiation (λ = 0.15418 nm) over the angular region 2θ between 5° and 40°. Compression-molded film samples were crystallized from the melt at 220 °C at cooling rates of 5 °C/min in a Mettler FP90/FP82 HT temperature cell (Mettler-Toledo SAE, Barcelona, Spain).

#### 2.2.3. Differential Scanning Calorimetry (DSC)

The non-isothermal crystallization studies were carried out using a Perkin Elmer DSC7/7700 differential scanning calorimeter (Perkin-Elmer España SL, Madrid, Spain) under a nitrogen purge. The instrument was calibrated for temperature and heat flow using high-purity indium and zinc standards, and the data were evaluated by using the DSC-7/UNIX program. A tau lag calibration of the instrument for different heating rates was performed using indium. The experimental and theoretical procedures used in this study were similar to those employed in our previous publication on PLLA/1D-WS_2_ [17]. The samples were first heated to 225 °C and held at this temperature for 5 min to erase their thermal history. Afterwards, cooling cycles from the melt were then undertaken for each sample at cooling rates (φ) of 1, 2, 5, 10 and 20 °C/min. The heat that evolved during the non-isothermal crystallization was recorded as a function of temperature. The crystallization peak temperature (*T_p_*) was determined from the minimum of the crystallization exotherm observed during the cooling scan. The apparent crystallization enthalpy was determined as the area below the transformation curve, taking as the upper and lower limits as the corresponding deviations in the baseline, crystallinity was calculated as follows:

(1) $$(1-\lambda )=\frac{\Delta H_{c}}{\Delta H_{m}^{0}}$$

where Δ*H_c_* is the crystallization enthalpy and Δ$H_{m}^{0}$ is the enthalpy of melting for perfect crystals (93 J/g) [26].

#### 2.2.4. Biodegradation Tests

The bacterial degradation of PLLA and the PLLA/2D-WS2 nanocomposite films was performed using the actinobacteria, *Lentzea waywayandensis* (DSM 44232) obtained from DSMZ-German Collection of Microorganisms and Cell Cultures GmbH. For this, sterilized films (5 mm × 5 mm × 0.3 mm) were placed in Erlenmeyer flasks containing 90 mL of basal culture medium that were supplemented with 0.1% gelatin and 10 mL of the actinobacteria liquid culture prepared according to reported procedures [27]. The biodegradation tests of the nanocomposite films were carried out at 30 °C and 180 rpm using an orbital shaker (KS 4000 i control, IKA) for incubation periods of 7, 14 and 21 days. All the tests were carried out in duplicate with control tests also conducted in the absence of the microorganism.

## 3. Results

### 3.1. Morphology and Structure

**Figure 1:**
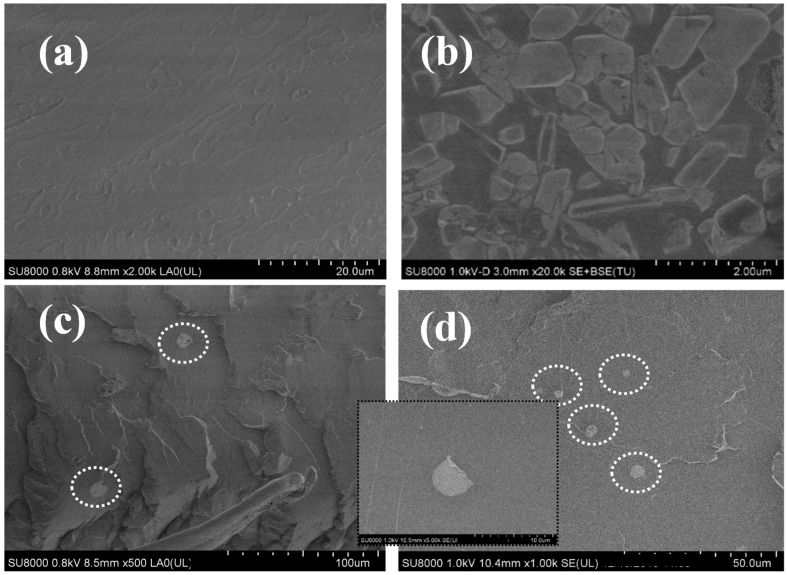
SEM micrographs of (**a**) poly(l-lactic acid) (PLLA), (**b**) tungsten disulfide nanosheets (2D-WS_2_) and PLLA/2D-WS_2_ nanocomposites with nanofiller loadings of (**c**) 0.5 and (**d**) 1.0 wt %. The white dashed circles represented the 2D-WS_2_.

It is well known that the dispersion and interfacial interaction between nanofillers and biopolymer matrices play a key role in the final properties of biopolymer nanocomposites [17]. SEM was employed to observe the micromorphology of the cryogenically fractured surfaces of PLLA, the nanocomposite films, and the neat WS_2_ nanosheets (Figure 1).

The morphological differences between PLLA and the PLLA/2D-WS_2_ nanocomposites are clearly visible. From Figure 1a, it can be seen that the fractured PLLA surface is comparatively smooth. In contrast, the fracture surfaces of the PLLA/2D-WS_2_ nanocomposites (Figure 1c,d) are relatively rough with the WS_2_ nanosheets being well dispersed, and are neither fully enclosed nor pulled-out from the PLLA matrix. This suggests that there is a strong interfacial interaction between the 2D-WS_2_ and the PLLA matrix encountered using simple shear force melt-blending. Typically, to achieve this, more elaborate methodologies have been employed, such as the synthesis of PLA/MoS_2_-NH_2_ nanocomposites via in situ ring-opening polymerization [28].

**Figure 2:**
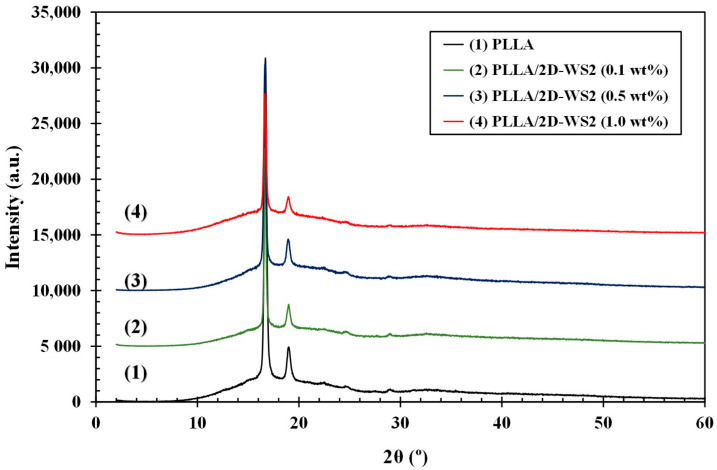
WAXS diffractograms of PLLA/2D-WS_2_ nanocomposites obtained at room temperature after cooling from the melt at 5 °C/min.

Wide-angle X-ray diffraction (WAXD) measurements were performed on the PLLA/2D-WS_2_ nanocomposites film samples with the same thermal history to be able to determine whether the addition of 2D-WS_2_ affected the PLLA crystalline structure (Figure 2). At room temperature, only the characteristic diffraction peaks of PLLA are seen, with the strongest visible diffraction peak being the characteristic (200)/(110) reflection of the α-form at 16.7° [17], implying that the 2D-WS_2_ nanoparticles have no impact on its crystalline structure. However, the crystallite size perpendicular to the diffraction characteristic (200)/(110) plane, D_200/110_, obtained from the room temperature diffractograms using well-known Scherrer formula, increases with the addition of 2D-WS_2_ (PLLA = 28.6 nm, PLLA/2D-WS_2_ (0.1 wt %) = 37.0 nm, PLLA/2D-WS_2_ 0.5 wt % = 33.1 and PLLA/2D-WS_2_ (1.0 wt %) = 29.9 nm). In particular, during the cooling process, PLLA crystals grow considerably, resulting in larger room temperature D_200/110_ values than the calculated for the pure matrix. On the other hand, the disappearance of the layered transition–metal dichalcogenide diffraction peaks suggests that the nanoparticle is highly exfoliated and/or the PLLA is well intercalated within the WS_2_ sheets [9,10].

### 3.2. Non-Isothermal Crystallization

**Figure 3:**
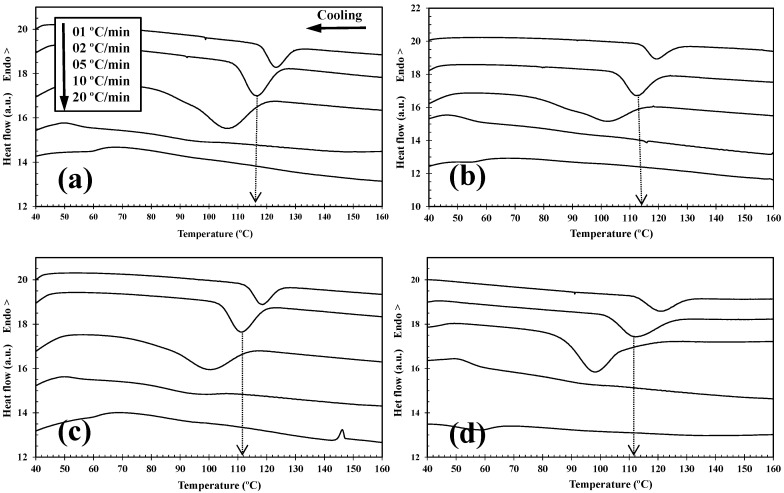
Differential Scanning Calorimetry (DSC) melt-crystallization thermograms of (**a**) PLLA and PLLA/2D-WS_2_ nanocomposites with nanofiller loadings of (**b**) 0.1, (**c**) 0.5, and (**d**) 1.0 wt % obtained at the cooling rates indicated.

The non-isothermal crystallization behavior of PLLA and the PLLA/2D-WS_2_ nanocomposites was investigated as this corresponds to the type of temperature changes that might occur in industrial applications. Figure 3 shows the effect of cooling rate and 2D-WS_2_ concentration on the non-isothermal crystallization behavior of the PLLA/2D-WS_2_ nanocomposites with the specific crystalline parameters of all samples listed in Table 1.

From the previous curves, useful parameters, such as the peak temperature (*T_p_*) and crystallinity (1-λ)_c_ as a function of crystallization temperature, can be obtained to describe the non-isothermal crystallization behavior of the tested materials. It can be seen that PLLA manifests slow crystallization on cooling from the melt, and it does not crystallize at a cooling rate of 10 °C/min or faster. Additionally, as the cooling rate increases, the crystallization exotherm broadens and shifts to lower temperatures for both the PLLA and PLLA/2D-WS_2_. This indicates that at slower cooling rates, a larger proportion of the tested semicrystalline polymers spent more time within a temperature range sufficient to promote chain segment mobility and crystal growth. With the increase in the cooling rate, the crystallization of the composite material gradually decreased. This is because the frozen molecular chain segments prevented the crystallization of PLA when the cooling rate was too fast. Furthermore, for a given cooling rate, the *T_p_* of PLLA/2D-WS_2_ was lower than that of pure PLLA, as shown in Figure 3, indicating that the addition of 2D-WS_2_ into PLLA decreased its rate of crystallization. This is because the surface of the WS_2_ nanosheets could not easily adsorb the PLLA chain segments, which would greatly hinder crystal growth. In particular, when the interparticle free space becomes smaller than the characteristic extended length of the polymer molecule, nanoparticles impede crystallization due to confinement effects. Based on the findings from the work of Jabbarzadeh, equations for critical particle size or volume fraction that led to this confinement-induced retardation of crystallization were proposed [19].

**Figure 4:**
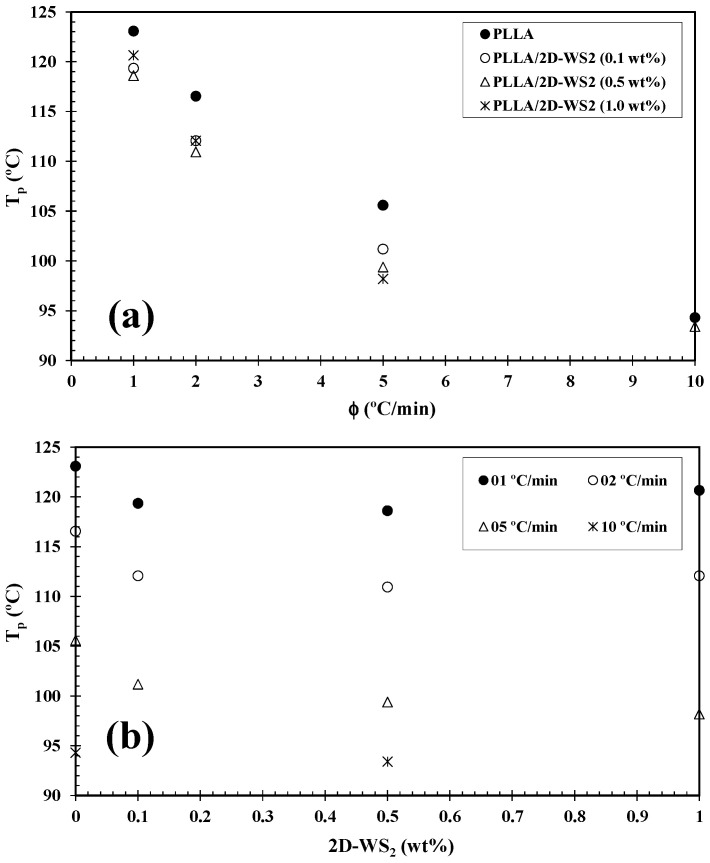
Variation of the crystallization peak temperature (*T_p_*) for PLLA/2D-WS_2_ nanocomposites with (**a**) cooling rate and (**b**) composition.

**Figure 5:**
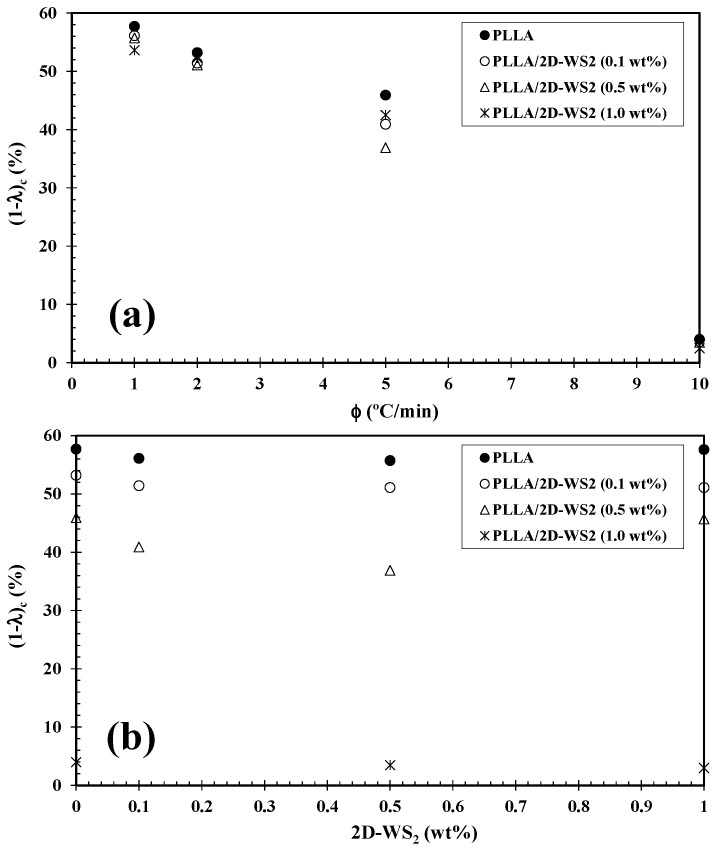
Variation of the crystallinity (1-α)_c_ for PLLA/2D-WS_2_ nanocomposites with (**a**) cooling rate and (**b**) composition.

For more clarity, Figure 4 summarizes the variation of *T_p_* with cooling rate and composition. In particular, as the addition of 2D-WS_2_ reduces the crystallization temperature of PLLA, it would imply that the nucleation of PLLA crystals is retarded by the WS_2_ nanosheets. This observation is reproducible for nanocomposites crystallized at different cooling rates. In contrast, 1D-WS_2_ nanotubes have been shown to accelerate the PLLA crystallization process via heterogeneous nucleation [17]. Such differences suggest that the nanoparticle shape plays a fundamental role in PLLA crystallization. In a similar manner to the 2D-WS_2_ nanosheets, the addition of Cloisite 30B (a organically modified montmorillonite [28]) to PLA was also found to retard its crystallization process. This was reported to be due to the good interfacial energy between the PLA matrix and the modifier used in Cloisite 30B hindering the PLA chain-folding process needed for crystallization. As such, it suggests that highly compatible clays dispersed within the polymer matrix can hinder the interchain interactions necessary for crystal nuclei formation. This discrepancy is likely related to several factors, including the difference in the thermal conductivity of the filler and polymer matrix, the nucleation efficiency (NE) of the filler, its state of dispersion within the matrix, and the potential existence of mechanisms of interfacial crystallization such as epitaxy and transcrystallization [29,30,31,32]. NE is strongly dependent on the nanofiller morphology, its surface energy, roughness, and crystalline structure as well as on the filler ability to form the critical nucleus [16,17,33,34]. Furthermore, the dependence of crystallinity (1-λ)_c_ of PLLA and its 2D-WS_2_ nanocomposites as a function of cooling rate (Figure 5a) closely mirrors the *T_p_* trends previously mentioned. This is expected, as at slower cooling rates, the polymer chains have more time to organize into crystalline domains with fewer defects and thus will present a higher (1-λ)_c_. However, the crystallinity value obtained from the crystallization exotherm of PLLA appears unchanged with the addition of 2D-WS_2_, particularly at low cooling rates (Figure 5b).

It is well-known that polymer crystallization releases a significant amount of heat, making DSC the preferred method for measuring overall crystallization kinetics. The measured rate of heat release is assumed to be proportional to the macroscopic rate of crystallization:

(2) $$\frac{dQ}{dt}=Q_{c}\frac{dx}{dt}$$

where *Q_c_* is the measured heat of crystallization calculated by integration of the DSC peak. The values of *Q_c_* can further be used to determine the crystallization rate (*dx*/*dt*) as well as the extent of the melt conversion:

(3) $$x\left(t\right)=\frac{1}{Q_{c}}\overset{t}{\underset{0}{\int }}\frac{dQ}{dt}dt$$

**Figure 6:**
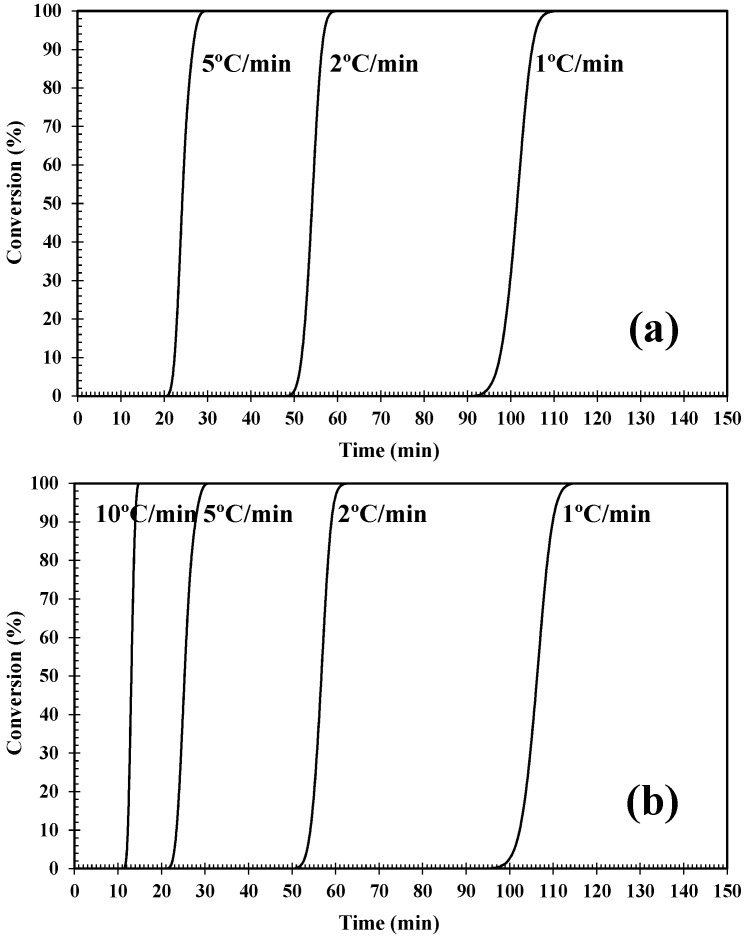
Plots of degree of conversion (x) vs. time for (**a**) PLLA and (**b**) PLLA/2D-WS_2_ (0.5 wt %) crystallized non-isothermally at various cooling rates.

The value of *x*(*t*) varies from 0 to 1 and represents the degree of conversion. The transformation from temperature to time is performed using a constant cooling rate *φ*:

(4) $$t=\frac{T_{0}-T}{\varphi }$$

where *T* is the temperature at time *t* and *T*_i_ is the temperature at the start of crystallization. Figure 6 shows typical conversion curves at various cooling rates for the PLLA/2D-WS_2_ nanocomposites. The conversion curves shift over to longer times with decreasing cooling rates, suggesting that the diffusion of PLLA becomes very difficult for melt crystallization. In order to quantitatively describe the evolution of crystallinity during non-isothermal crystallization, a number of models have been proposed in the literature. In this investigation, the Lui model was tested.

### 3.3. Lui Model

**Figure 7:**
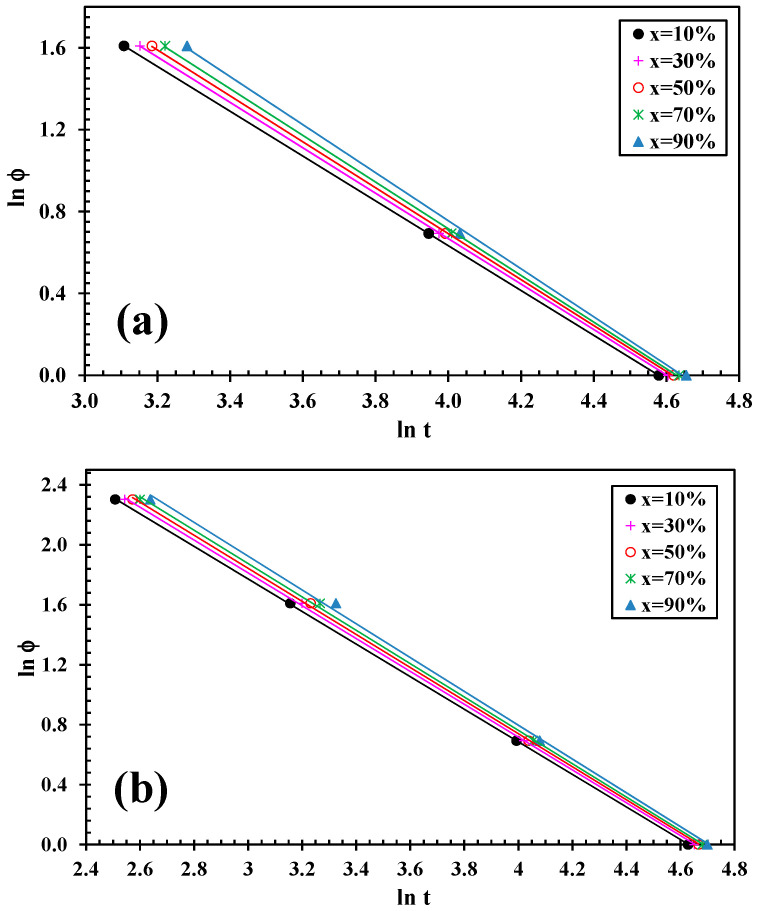
Lui plots for melt crystallization of (**a**) PLLA and (**b**) PLLA/2D-WS_2_ (0.5 wt %).

A convenient approach adopted to describe the non-isothermal crystallization was the Liu model [35]. By combining the Avrami [36,37,38] and Ozawa [39] equations, the Liu model has been proved to be suitable and convenient to handle the non-isothermal crystallization of polymer nanocomposites [40]. As the degree of conversion (x) is related to the cooling rate *φ* and the crystallization time *t* (or temperature *T*), the relation between *φ* and *t* could be defined for a given degree of conversion. Consequently, the kinetic equation for non-isothermal crystallization was derived:

(5) lnφ=lnf(T)−αlnt

where *f*(*T*) = [k’(T)/k]^1/m^ refers to the value of cooling rate chosen at a unit crystallization time, when the system has a certain degree of crystallinity, and α is the ratio of the Avrami exponents to Ozawa exponents (i.e., α = n/m). According to Equation (5), at a given degree of conversion, the plot of ln *φ* vs. ln *t* gives a series of lines, as can be seen in Figure 7. This indicates that the Lui model provides a satisfactory description for the non-isothermal crystallization for PLLA/2D-WS_2_ nanocomposites. The kinetic parameters, ln *f*(*T*) and *α*, which are derived from the slope and the intercept of those lines respectively, are listed in Table 1.

The *f*(*T*) values increased rapidly with an increase in the relative crystallinity of all samples. However, the *f*(*T*) value of PLLA/2D-WS_2_ is smaller than that of neat PLLA, meaning that the addition of 2D-WS_2_ to PLLA needs a higher cooling rate to approach an identical degree of crystalline transformation. In other words, the rate of crystallization of the PLLA/2D-WS_2_ nanocomposites is lower than that of PLLA. This is also in good agreement with the results observed in Figure 3 and Figure 4b. In addition, the values of the parameter α are nearly constant (1.1 to 1.7), indicating that the mechanism of nucleation and growth is approximately the same for both PLLA and the PLLA/2D-WS_2_ nanocomposites.

### 3.4. Effective Energy Barrier

**Figure 8:**
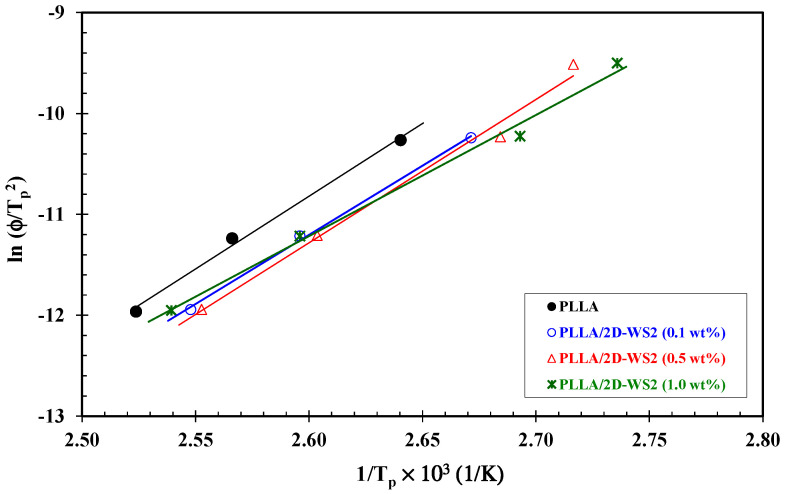
Kissinger plots for evaluating effective energy barrier of PLLA/2D-WS_2_ nanocomposites.

There are many mathematical approaches to evaluate the crystallization activation energy, or effective energy barrier, Δ*E* of the crystallization process. The approach proposed by Kissinger is used in this study [41]. Considering the variation of the crystallization peak temperature *T_p_* with cooling rate *φ*, the Δ*E* could be determined as follows:

(6) $$ln\left(\frac{\varphi }{T_{p}^{2}}\right)=Constant-\frac{\Delta E}{RT_{p}}$$

where *R* is the universal gas constant. The calculated values of activation energy (Figure 8) are given in Table 1. It can be concluded that the addition of 2D-WS_2_ caused a decrease in the Δ*E*, making the molecular chains of PLLA more difficult to crystallize. As such, it is verified again that the 2D-WS_2_ do not nucleate PLLA.

### 3.5. Biodegradation Tests

**Figure 9:**
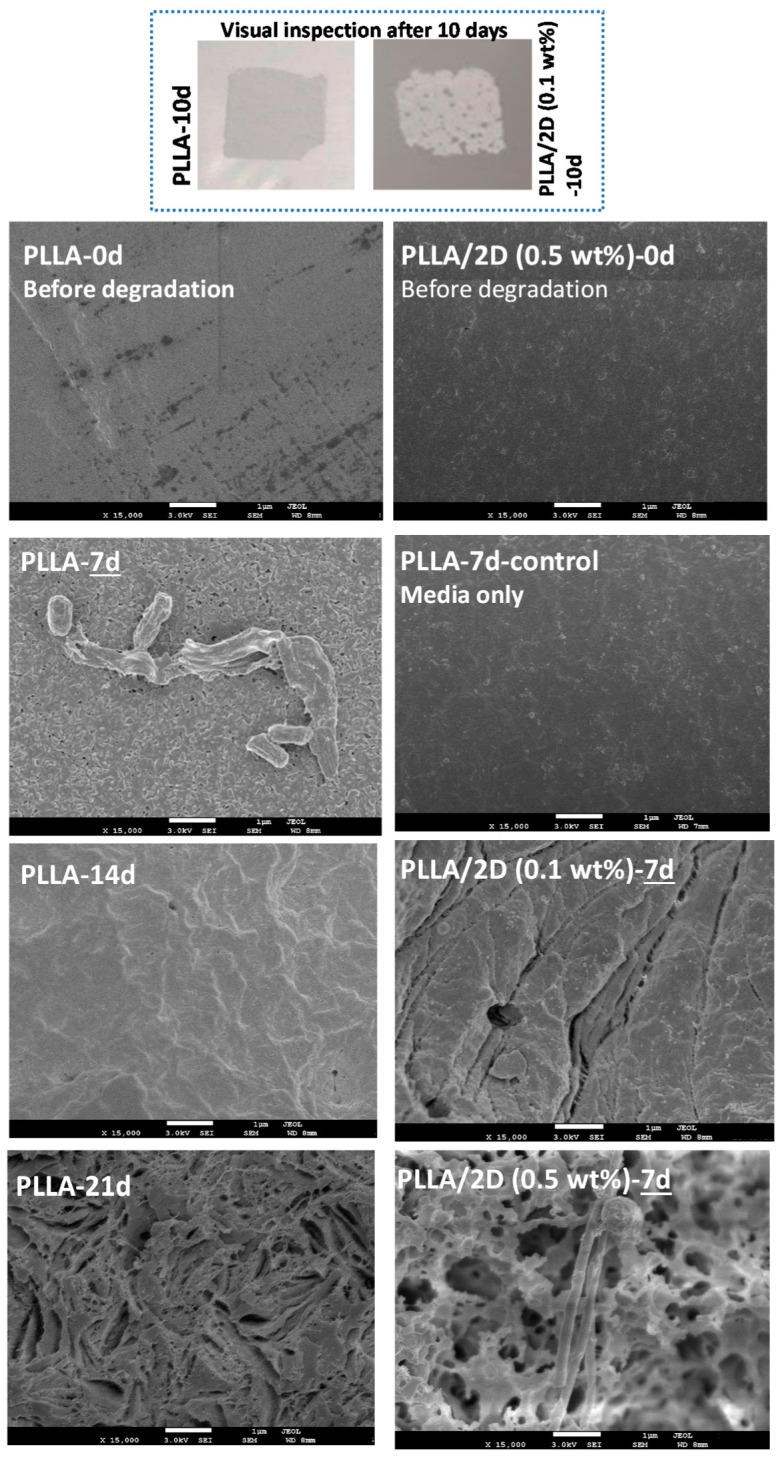
Optical and SEM micrographs of PLLA and PLLA/2D-WS_2_ (0.5 and 1.0 wt %) nanocomposites obtained at the incubation times indicated.

Polymer degradation is associated with changes in characteristics, such as the color and surface morphology. Effects used to describe degradation include roughening of the surface, the formation of holes or cracks, de-fragmentation, changes in color, or the formation of bio-films on the surface. The PLLA films, which were initially transparent and amorphous, became a translucent white after 7 days of incubation in the presence of *Lentzea waywayandensis*. After 21 days, the surface of the neat PLLA changed to a yellowish–dark brown color, which is caused by water permeation and microorganism activity. Figure 9 shows the surface morphology of PLLA and the 2D-WS_2_ nanocomposite films under SEM. Before the degradation trials, the surface of neat PLLA and the PLLA/2D-WS_2_ nanocomposites was smooth. After 7 days, the neat PLLA did not present any significant surface changes in the presence of the actinobacteria, and at 14 days, only the surface roughness had increased. However, in the case of the PLLA/2D-WS_2_ nanocomposites after 7 days, their surfaces exhibited the presence of obvious cracks and clearly showed considerable degradation likely as a result of enhanced PLLA hydrolysis and microorganisms activity. With the addition of WS_2_ nanosheets, the cracks and voids became substantially deeper and larger and thereby suggest more surface erosion during a shorter incubation period. This bulk erosion hydrolytic degradation process is comparable to that observed for PLA and PLA/T_i_O_2_ nanocomposite systems [19].

In summary, this study is the first step to exploring PLLA nanocomposites degradation using a promising actinobacteria (*Lentzea waywayandensis*) and understanding how degradation changes based on the addition of 2D-WS_2_. In future work, we will focus on both the regulatory mechanisms involved in actinobacteria PLLA degradation as well as the enzymes acting upon the polymer. The combination of physical, chemical, and biochemical modifications to the active enzymes together with controlling regulatory mechanisms could lead to more efficient polymer degradation.

## 4. Conclusions

In this work, the dispersion of WS_2_ nanosheets in a PLLA matrix was achieved via melt processing, a simple, scalable, cost-effective and ecologically method. SEM and WAXS demonstrated that the 2D-WS_2_ were well dispersed, intercalated, and/or exfoliated in the PLLA matrix. DSC analysis revealed that the non-isothermal crystallization behavior of the PLLA/2D-WS_2_ nanocomposites was strongly dependent on the 2D-WS_2_ content and cooling rate. In particular, the incorporation of 2D-WS_2_ at a relatively low concentration induced a significant reduction in the crystallization rate of PLLA due to the physical barrier action of the nanosheets while maintaining the crystal structure of PLLA. Furthermore, the method developed by Liu at al. could successfully describe the complex crystallization kinetics of the PLLA/2D-WS_2_ nanocomposites occurring during continuous cooling. The parameter *f*(*T*), which has a physical and practical significance, decreased with 2D-WS_2_ loading, indicating that the addition of the nanoparticles hindered the PLLA polymer chain transportation to the crystal growth front. In the same manner, the effective energy barrier governing the non-isothermal crystallization confirms the evident decrease in the PLLA crystallization rate in the PLLA/2D-WS_2_ nanocomposites. Finally, biodegradation analysis showed that the incorporation of the 2D-WS_2_ nanoparticles into the typically poorly biodegradable PLLA matrix facilitated its degradation in the presence of actinobacteria (*Lentzea waywayandensis*). 

## Figures and Tables

**Table 1:** Crystallization parameters of the PLLA/2D-WS_2_ nanocomposites.

2D-WS_2_	*φ* (°C/min)	*T_p_* (°C)	(1−λ) ^c^ (%)	*x*^a^ (%)	α ^b^	*f*(*T*) ^b^	Δ*E* ^c^ (kJ/mol)
0	1	123.1	57.7	10	1.09	5.01	−119.9
	2	116.5	53.2	30	1.11	5.1	
	5	105.6	45.9	50	1.12	5.18	
	10	94.3	4	70	1.14	5.27	
	20	-	-	90	1.17	5.45	
0.1	1	119.3	56.1	10	1.1	5.08	−114.1
	2	112.1	51.4	30	1.11	5.13	
	5	101.2	40.9	50	1.13	5.24	
	10	-	-	70	1.15	5.35	
	20	-	-	90	1.18	5.5	
0.5	1	118.6	55.7	10	1.09	5.03	−118.2
	2	110.9	51.1	30	1.1	5.1	
	5	99.4	36.9	50	1.1	5.16	
	10	93.4	3.5	70	1.11	5.21	
	20	-	-	90	1.13	5.31	
1	1	120.7	57.6	10	1.09	5.01	−99.9
	2	112.1	56.2	30	1.11	5.11	
	5	98.2	45.7	50	1.11	5.17	
	10	-	3	70	1.12	5.22	
	20	-	-	90	1.12	5.28	

^a^ The corresponding values of volume fraction are 0.01, 0.13, and 0.67%; ^b^ Crystallization parameters calculated using Liu’s equation. ^c^ Effective energy barrier calculated using Kissinger’s equation.
